# Impact of Early Versus Late Diagnosis on Disease Progression in Cystinosis

**DOI:** 10.1016/j.ekir.2024.10.037

**Published:** 2025-03-04

**Authors:** Mark R. Benfield

**Affiliations:** 1 Pediatric Nephrology of Alabama, PC, Cullman, Alabama, USA

## Introduction

**Figure 1:**
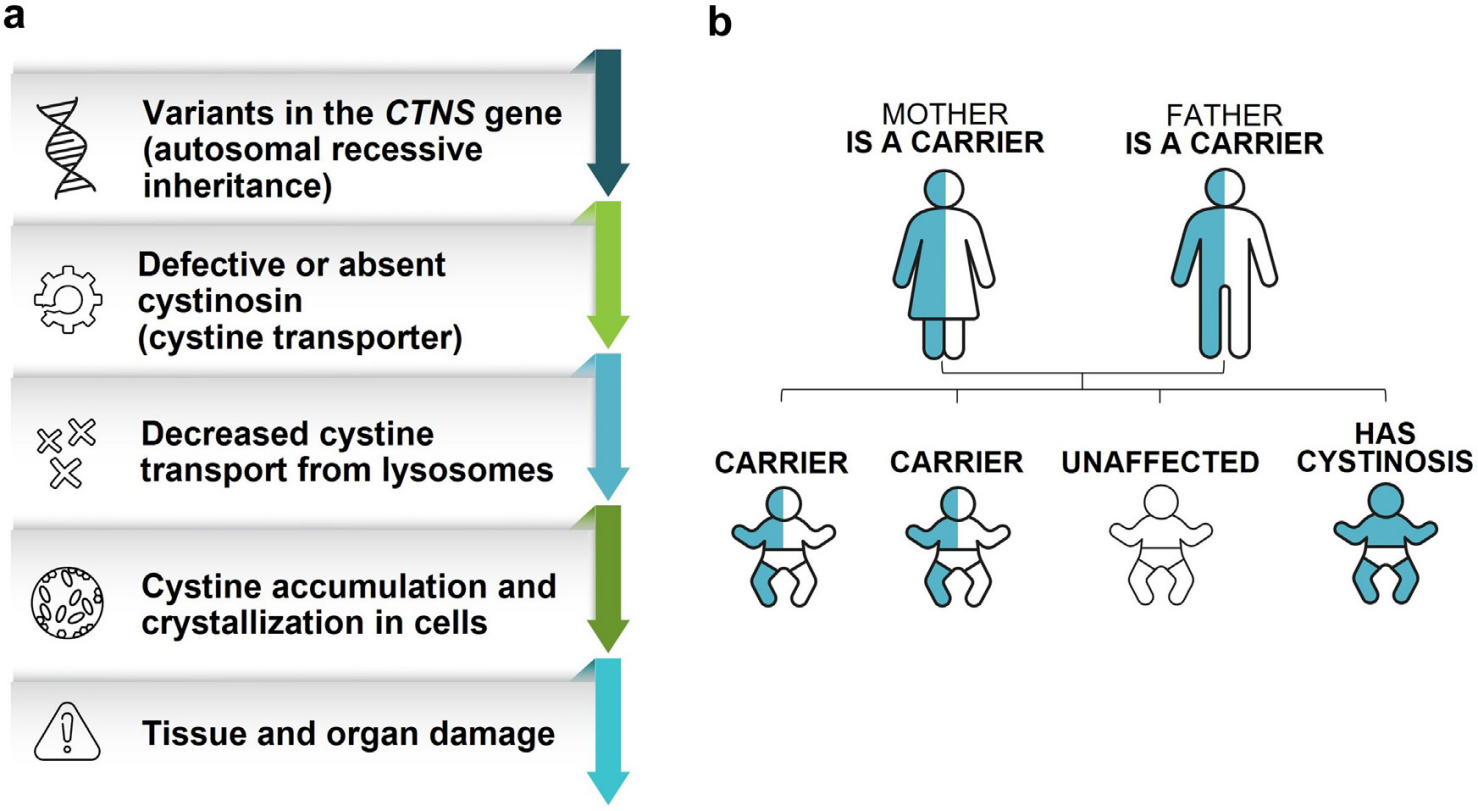
Overview of cystinosis. (a) Cystinosis is caused by variants in the *CTNS* gene, which encodes for the lysosomal cystine transporter cystinosin. Defective or absent cystinosin causes cystine accumulation within cells, ultimately leading to progressive tissue and organ damage throughout the body.^7^ (b) Cystinosis is an autosomal recessive condition, requiring the inheritance of 2 gene variants. With each pregnancy for parents who both carry 1 copy of a nonworking *CTNS* gene variant, there is a 25% chance the child will have cystinosis, 50% chance the child will be a carrier of the condition, and 25% chance the child will be unaffected.^8^

Nephropathic cystinosis is an ultrarare autosomal recessive lysosomal storage disorder, characterized by progressive cystine accumulation (Figure 1).^1^ The kidneys are the first and most seriously affected organs; as the disease progresses, nearly all body systems are affected.^2^^,^^3^ Treatment with oral cysteamine has transformed the natural history of cystinosis, and recent studies have highlighted the positive effects of initiating cysteamine treatment as early as possible to support optimal outcomes.^2^^,^^4^, ^5^, ^6^ Cystinosis is typically diagnosed in the second year of life, delaying the time to treatment initiation.^5^^,^^6^ Here, I describe the diagnosis, management, and disease progression of cystinosis in 2 siblings who are presumed to carry the same *CTNS* variants and highlight the potential differences in outcomes relative to age at diagnosis.

## Case Presentation

### Initial Presentation and Diagnosis

#### Patient A

Patient A is a 13-year-old girl who was diagnosed with nephropathic cystinosis in childhood (Table 1). She presented with growth failure and recurrent episodes of severe polyuria, polydipsia, vomiting, lethargy, and hypokalemia from the age of 1 to 3 years, requiring multiple hospital admissions for intravenous fluid resuscitation. Further workup during an episode at the age of 3 years led to her cystinosis diagnosis via slit-lamp eye examination and elevated white blood cell cystine level.

**Table 1:** Comparison of the clinical course between patients A and B

Presentation and diagnosis			
	Age at diagnosis	Symptoms at diagnosis	Diagnostic testing
Patient A	3 yrs	• Fanconi syndrome • Recurrent episodes of polyuria, polydipsia, vomiting, lethargy, hypokalemia • Corneal cystine crystals • Growth failure	• Slit-lamp eye examination • WBC cystine level testing
Patient B	18 mo	• Mild Fanconi syndrome • Growth failure • Genu valgum of uncertain etiology	• WBC cystine level testing


Growth trends			
	**Time point**	**Weight**	**Height**
Patient A	7 yrs 13 yrs	21.5 kg (50th percentile) 57.0 kg (75th percentile)	112 cm (10th percentile) 158 cm (50th percentile)
Patient B	5 yrs 11 yrs	19.5 kg (50th percentile) 37.0 kg (50th percentile)	107 cm (25th percentile) 142 cm (25th percentile)


**WBC cystine level testing**			
	**Diagnosis (mixed leukocytes; RR: 0.2 nmol ½ cystine/mg protein**^a^**)**	**Last observed value (granulocytes; RR <1.9 nmol ½ cystine/mg protein)**	**Trends over time**
Patient A	3.21	1.32	Stable, with levels below target range for the past 7 yrs
Patient B	3.6	1.8	Stable, with levels below target range for the past 7 yrs


**Laboratory studies**			
	**Kidney function (last observed value)**		
Patient A	Aged 13 yrs eGFR: 28 ml/min per 1.73 m^2^ Creatinine: 2.3 mg/dl (RR: 0.4–1.0) Protein-to-creatinine ratio: 1.8		
Patient B	Aged 11 yrs eGFR: 57 ml/min per 1.73 m^2^ Creatinine: 1.0 mg/dl (RR: 0.4–1.0)		


**Medications**			
	**Age at cysteamine initiation**	**Current cysteamine dosing**	**Other current medications/supplements**
Patient A	3 yrs (after diagnosis)	DR cysteamine 1050 mg Q12H Cysteamine eye drops (1 drop per eye), every waking hour	Potassium chloride ER 40 mEq QID Sodium bicarbonate 650 mg BID Indomethacin 25 mg BID Omeprazole 20 mg QD Calcitriol 0.25 mcg 3 times weekly Levocarnitine 250 mg BID Ferrous sulfate 325 mg BID Somatropin 1.2 mg QD
Patient B	18 mo (after diagnosis)	DR cysteamine 900 mg Q12H Cysteamine eye drops (1 drop per eye), every waking hour	Phospha 250 Neutral 250 mg QD Potassium citrate ER 40 mEq BID Levocarnitine 250 mg QD Ferrous sulfate 325 mg QD Somatropin 1.8 mg QD

BID, twice daily; DR, delayed-release; eGFR, estimated glomerular filtration rate; ER, extended-release; Q12H, every 12 hours; QD, once daily; QID, four times daily; RR, reference range; WBC, white blood cell.

a Normal mixed leukocyte cystine level in individuals without cystinosis is <0.2 nmol ½ cystine/mg protein; target mixed leukocyte cystine level in individuals with cystinosis is <1.0 nmol ½ cystine/mg protein.

#### Patient B

Patient B is the 11-year-old brother of patient A. He developed mild Fanconi syndrome with electrolyte imbalance, failure to thrive, and genu valgum of uncertain etiology at the age of 18 months. Because of his symptoms and his sister’s recent diagnosis, white blood cell cystine level testing was done, which confirmed that he also has cystinosis (Table 1). Genetic testing was not performed for either sibling.

### Management Course and Multidisciplinary Care

#### Management Course – Patient A

After her diagnosis, patient A was started on fluid and electrolyte supplementation and immediate-release cysteamine bitartrate (Cystagon; Viatris Inc, Canonsburg, PA). The patient experienced severe vomiting with the prescribed immediate-release cysteamine dosage and was unable to achieve significantly reduced white blood cell cystine levels. She continued to experience growth failure and episodes of dehydration.

Rather than choosing gastrostomy tube placement, the patient’s mother elected to give fluids and electrolytes every 1 to 2 hours during the daytime, with rapid repletion of fluids each morning. At approximately the age of 4 years, patient A was switched from immediate-release to delayed-release cysteamine bitartrate (Procysbi; Amgen Inc, Thousand Oaks, CA), which allowed for slight stabilization in clinical status, with improved weight gain and markedly decreased white blood cell cystine levels within target range. She was started on growth hormone at approximately 5 years of age. The patient continued to require 10 to 12 L of fluid and 18 mEq/kg of potassium chloride per day and had recurrent episodes of dehydration with lethargy. Indomethacin was added to her regimen, which resulted in significantly improved fluid and electrolyte balance and a decrease in hospitalizations. This patient currently has stage 3b chronic kidney disease.

#### Management Course – Patient B

After his diagnosis, patient B initiated fluid and electrolyte supplementation and delayed-release cysteamine. He experienced markedly improved fluid and electrolyte balance, resolution of renal osteodystrophy, and appropriate weight gain. He was also started on growth hormone in childhood to support height velocity. He has had no hospitalizations, requires only modest doses of electrolyte replacement therapies, and currently has stage 2 chronic kidney disease.

#### Multidisciplinary Coordination

Both siblings visit the nephrology clinic every 3 months. Patient A also sees multiple specialists, including a kidney transplant team, ophthalmologist (eye progression), gastroenterologist (medication tolerance, gastrointestinal symptoms), and orthopedic specialist (metabolic bone disease). Patient B does not yet require other specialist visits. Both siblings undergo yearly screening for extrarenal complications; none have been identified thus far.

### Development and Psychosocial Considerations

The patients live with their mother and several foster children. Due to her severe disease, patient A was homeschooled from 5 to 8 years of age. She began attending public school at the age of 9 years, as her condition improved with the addition of indomethacin. However, she misses school or visits the school nurse 1 to 3 times per week and is unable to participate in physical education or other school-related activities. Patient B has always attended public school and is able to participate in all school-related activities with only rare absences. Both patients are performing well academically at their expected grade levels, although they have not undergone formal neurocognitive testing.

## Results

As these 2 siblings get older, it is anticipated that patient A will continue having challenges that her younger brother may not experience. Although a preemptive transplant is being considered, patient A will likely require bilateral nephrectomy and short-term dialysis before transplant because of her continued fluid and electrolyte loss. In addition, patient A will soon begin planning for her eventual transition to adult care. This transition will be especially challenging because of the need for significant caregiver involvement and the scarcity of adult clinicians, including subspecialists, who are willing to provide necessary support for patients with cystinosis and severe disease. Patient B’s development and disease progression will continue to be monitored, with a focus on supporting his self-management skills and encouraging continued adherence to cysteamine therapy.

## Discussion

Historic cohort studies have shown the importance of early diagnosis, prompt treatment initiation, and long-term cysteamine adherence in delaying the need for kidney transplant and limiting the effects of extrarenal manifestations.^7^^,^^9^ A study of 86 adults with cystinosis demonstrated a significantly decreased incidence and later onset of kidney failure, diabetes, hypothyroidism, and neuromuscular complications in individuals who started cysteamine treatment before the age of 5 years compared with untreated patients.^9^ Those who began taking cysteamine after the age of 5 years did not have a reduction in incidence or delayed onset of kidney failure compared with untreated patients, highlighting the importance of early treatment initiation.^9^ More recent publications have reported on the impact of even earlier diagnosis and cysteamine initiation before the age of 2 months in a small cohort of 6 patients.^5^^,^^6^ None of the patients in this early treated group had reached kidney failure by a mean age of 8.2 years (range: 1.2–17.2 years).^6^ In addition, these patients exhibited nearly normal growth, with only 1 patient receiving recombinant human growth hormone.^5^ Although these studies included a small number of patients, researchers hypothesized that it may be possible to ameliorate kidney failure by treating patients with cystinosis from birth.^5^^,^^6^

**Figure 2:**
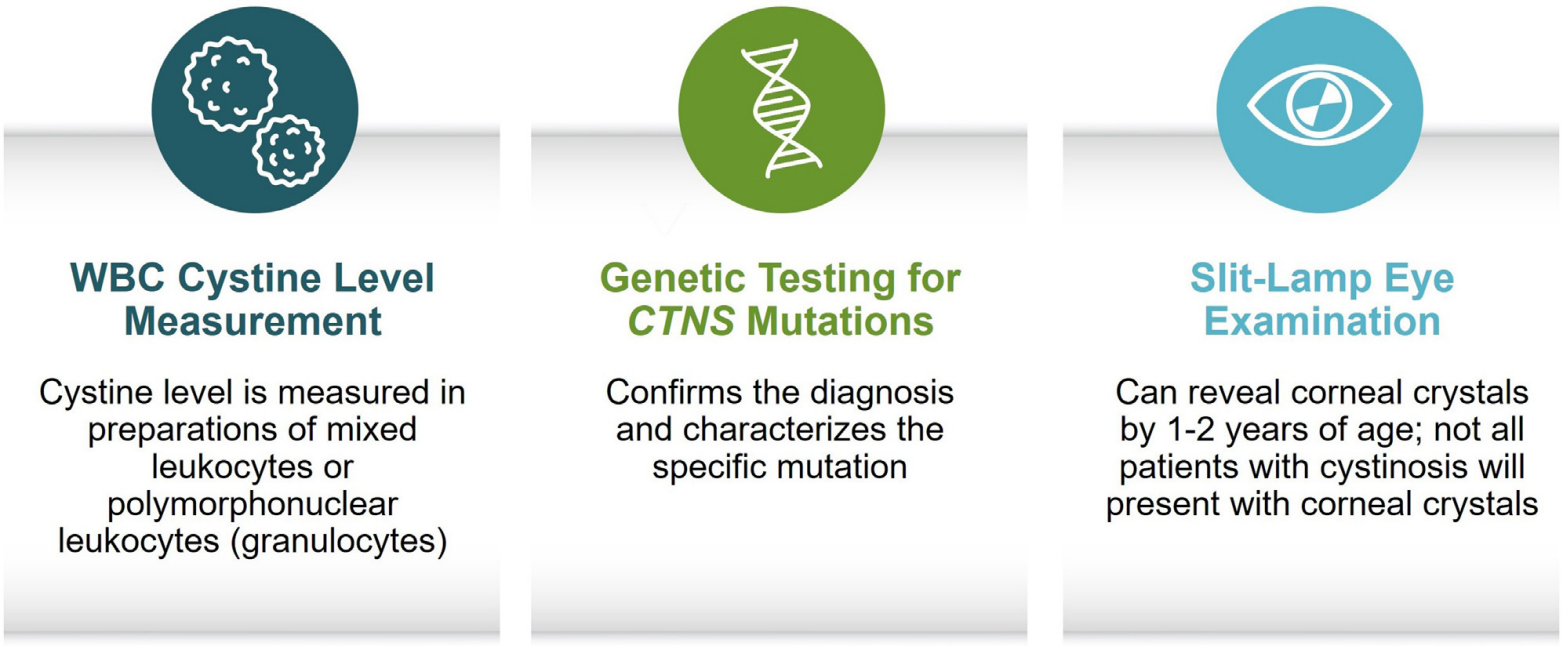
Current methods for the diagnosis of cystinosis. Cystinosis is generally suspected based on signs or symptoms suggestive of the disease.^1^ Once suspected, the diagnosis can be confirmed by WBC cystine level measurement, genetic testing for mutations in the *CTNS* gene, or slit-lamp eye examination, if the patient is old enough for corneal crystals to be present.^1^^,^^4^^,a^ ^a^The feasibility of including cystinosis in newborn screening programs is being evaluated.S1 WBC, white blood cell.

Although these results are encouraging, it is difficult to achieve such early diagnosis without implementing widespread newborn screening for cystinosis (Figure 2). In 2018, Germany implemented a pilot program to include molecular genetic screening for cystinosis, as part of their existing newborn screening initiative.S1 In this program, 2 patients were identified before symptom onset, allowing the opportunity for earlier treatment initiation.S1 Even in families with a known history of cystinosis, a later diagnosis in one sibling may delay diagnosis for the younger sibling. In this case, it is unclear if patient A’s diagnosis at the age of 3 years delayed or possibly hastened patient B’s diagnosis. Regardless, the differences between the 2 patients are striking. Patient A experienced a complicated course with severe Fanconi syndrome, repeated hospitalizations, and the need for significant fluid and electrolyte supplementation. Her medical instability placed a significant burden on the family, and she required homeschooling for several years to maintain fluid and electrolyte balance. Patient B has had a straightforward medical course, limited to mild Fanconi syndrome and subsequent orthopedic abnormalities, and a relatively normal childhood. Although environmental and epigenetic factors cannot be ruled out, patient B started cysteamine when he was 1.5 years younger than his sister, which may have had a protective effect on his kidneys.

Although the use of indomethacin in cystinosis has been controversial because of its nephrotoxicity and gastrointestinal side effects, a large international cohort study did not find worsened kidney outcomes in patients who were treated with long-term indomethacin.^4^ To help manage her renal losses, patient A was started on indomethacin which improved her fluid and electrolyte status and allowed her to attend public school. She remains on indomethacin and has only experienced a mild decrease in glomerular filtration rate.

This case further supports the need for early diagnosis and cysteamine initiation to improve outcomes in patients with cystinosis (Table 2).

**Table 2:** Teaching points

Diagnosis of cystinosis
• Cystinosis should be suspected in pediatric patients who present with symptoms of Fanconi syndrome; diagnosis can be confirmed via genetic testing, WBC cystine level measurement, or slit-lamp eye examination
• Even in siblings, the clinical course of cystinosis can vary widely, which may be due to earlier diagnosis and treatment initiation in the younger sibling; environmental and epigenetic factors may also play a role
• Cystinosis places a high medical burden on patients and their caregivers; clinicians should be mindful of psychosocial and socioeconomic factors that may impact care and overall outcomes

WBC, white blood cell.

## Disclosure

MRB has participated in advisory boards and/or received honoraria from Amgen Inc (formerly Horizon Therapeutics plc).

## Patient Consent

Consent was obtained from the patients’ parents for this case report.

## Supplementary Material

Supplemental File (PDF) **Supplementary References**.
