# Supplementary material for: Impact of Early Versus Late Diagnosis on Disease Progression in Cystinosis
Source: Kidney Int Rep. 2025 Mar 4;10(3 Suppl):S779–83. doi: 10.1016/j.ekir.2024.10.037 (PMC11935125; doi:10.1016/j.ekir.2024.10.037)
Supplement: Supplemental File (PDF) — Supplementary References. [file mmc1.pdf]

SUPPLEMENTARY REFERENCES

S1. Hohenfellner K, Elenberg E, Ariceta G, et al. Newborn screening: review of its impact for cystinosis. Cells. 2022;11(7):1109. https://doi.org/10.3390/cells11071109
